# Occurrence of Deoxynivalenol, Nivalenol, and Their Glucosides in Korean Market Foods and Estimation of Their Population Exposure through Food Consumption

**DOI:** 10.3390/toxins12020089

**Published:** 2020-01-29

**Authors:** Sang Yoo Lee, So Young Woo, Fei Tian, Jeonghun Song, Herbert Michlmayr, Jung-Bok Kim, Hyang Sook Chun

**Affiliations:** 1 Advanced Food Safety Research Group, BK21 Plus, School of Food Science and Technology, Chung-Ang University, Anseong 17546, Korea; dm3822@naver.com (S.Y.L.); mochalatte9@naver.com (S.Y.W.); tianfei_real@163.com (F.T.); soko0914@naver.com (J.S.); 2 Department of Applied Genetics and Cell Biology, University of Natural Resources and Life Sciences, Vienna, (BOKU), 3430 Tulln, Austria; herbert.michlmayr@boku.ac.at; 3 Korea Advanced Food Research Institute, Uiwang 16001, Korea; amsam@daum.net

**Keywords:** deoxynivalenol, nivalenol, glucoside conjugate, market food, occurrence, exposure assessment

## Abstract

Major type B trichothecene mycotoxins, including deoxynivalenol (DON), nivalenol (NIV), and their respective glucoside conjugates, deoxynivalenol-3-β-D-glucose (DON3G) and nivalenol-3-β-D-glucose (NIV3G), are present in food products, such as cereals, legumes, and their processed products. Thus, here, DON, NIV, and their 3-β-D-glucosides were monitored in 506 Korean market foods, and exposure to these mycotoxins was estimated in the population consuming these foods. The accuracy and precision of our method, which simultaneously determined four toxins, were 80.1–106.5% and 0.3–12.4%, in four representative food matrices assessed. The incidences of DON, DON3G, NIV, and NIV3G among all food samples tested were 13%, 8%, 12%, and 5%, respectively. The glucoside conjugate with free toxin was found to have the maximum co-occurrence of 49%. The estimated daily intakes of DON, DON3G, NIV, and NIV3G through food intake under four different scenarios were 0.019–0.102, 0.004–0.089, 0.007–0.094, and 0.002–0.095 μg kg^−1^ body weight (b.w.) day^−1^, respectively, which are lower than the established health-based guidance values. Overall, our results suggest that the estimated exposure of the Korean population to type B trichothecenes, namely, DON, NIV, and their 3-β-D-glucoside conjugates, may not pose a potential health risk.

## 1. Introduction

The two major type B trichothecene mycotoxins, deoxynivalenol (DON) and nivalenol (NIV) are produced by different chemotypes of the *Fusarium* species [1] and they are often found to co-contaminate foods, such as cereals, legumes, and their processed products [2,3]. Among these mycotoxins, earlier studies focused on DON because of its more frequent natural occurrence than NIV, although a more frequent occurrence of NIV than DON has been documented in European and Asian countries, including Korea [4,5]. In Korea, DON and NIV have been detected in cereals (white rice, brown rice, and bran) at rates of 9.1–41.5% [6].

Recently, DON-3-glucoside (DON3G) and NIV-3-glucoside (NIV3G), major glucose conjugates of DON and NIV, also called masked or modified mycotoxins, have been identified as potent toxins because they are converted to free trichothecenes (DON and NIV) after hydrolysis during the digestive process in mammals [7,8,9]. These two glucoside conjugates often co-occur with free trichothecenes, because when plants are contaminated with free trichothecenes, the plants’ defense mechanisms attach polar groups, such as β-D-glucopyranoside, to hydroxyl groups of the free trichothecenes [10,11]. In fact, nearly 30% of cereals were found co-contaminated with DON3G and free toxins [12,13]. Thus, it is important to stringently monitor and manage free trichothecenes and their glucoside conjugates.

Cereals and legumes, such as white rice, brown rice, barley, wheat, maize, oat, sorghum, soybeans, red beans, peas, and others, are consumed frequently by Koreans and are found to be most frequently contaminated by type B trichothecenes [14,15,16]. Moreover, these contaminating trichothecenes remain in cereals and legumes even after heating or fermentation processing [17,18].

At a cellular level, DON inhibits ribosome binding and protein synthesis, which disrupts intracellular signaling and, ultimately, leads to cell death [19]. The acute toxic effects of DON include vomiting, diarrhea, and loss of appetite. Chronic exposure to DON may cause anorexia, delayed growth, immunotoxicity, and reproductive toxicity [20]. NIV is reported to be more toxic than DON, although it differs from DON only by an additional hydroxyl group [21,22].

To avoid the potential health risks after consuming DON, NIV, and their glucoside conjugates, legislation concerning tolerable daily intake (TDI) amounts of these toxins has been published. The Joint FAO/WHO Expert Committee in Food Additives (JECFA) established a maximum TDI of 1 μg kg^−1^ b.w. day^−1^ for the sum of DON, 3-ADON, and 15-ADON [23]. For NIV, a TDI of 0.4 μg kg^−1^ b.w. day^−1^ was established by the Food Safety Commission of Japan (FSCJ) [21]. Recently, the EFSA Panel on Contaminants in the Food Chain (CONTAM) suggested a “group TDI”, including glucoside conjugates, considering that glucosides are hydrolyzed to their free form in the intestinal tract [24,25].

DON, NIV, and their 3-β-D-glucoside conjugates are frequent contaminants in cereals, legumes, and processed foods consumed in Korea. However, the occurrence of trichothecenes and their glucoside conjugates in these foods and the estimated exposure of the population to these mycotoxins are limited. In this study, DON, NIV, and their glucoside conjugates were simultaneously analyzed in commercial foods that are vulnerable to contamination with trichothecenes. Furthermore, the exposure of the Korean population to these four toxins through food intake was estimated.

## 2. Results and Discussion

### 2.1. Accuracy and Precision of the High-Performance Liquid Chromatography (HPLC) Method

The recovery of simultaneously determined DON, NIV, and their glucosides is mentioned in Table 1. The recovery of DON, DON3G, NIV, and NIV3G in baby food, selected as a representative matrix of solid flour, was 87.4%, 86.5%, 103.3%, and 90.6% (2 × limit of quantification (LOQ)) and 84.7%, 82.9%, 90.8%, and 88.2%, respectively (5 × LOQ). In soybean paste (solid-paste matrix), the recovery was 95.8%, 84.5%, 85.4%, and 98.3% (2 × LOQ) and 96.7%, 89.7%, 84.9%, and 94.1%, respectively (5 × LOQ). In sorghum (solid-colored matrix), the recovery was 103.9%, 87.1%, 98.2%, and 88.9% (2 × LOQ) and 87.9%, 86.5%, 80.1%, and 80.6%, respectively (5 × LOQ). In Korean rice wine (liquid matrix), the accuracy was 100%, 103%, 95%, and 106.5% (2 × LOQ) and 104.7%, 96.2%, 100.1%, and 105.8%, respectively (5 × LOQ). The precision ranged from 0.3% to 12.4% in baby food, 2.6% to 12.9% in soybean paste, 1.9% to 9.9% in sorghum, and 2% to 3.4% in Korean rice wine.

The accuracy and precision were reliable in all matrices per the criteria of the European Commission Regulation 401/2006/EC. The accuracy of recovery of the four toxins in baby food and Korean rice wine ranged from 82.9% to 106.5%, similar to the results of our previous study, 78.7% to 106.5% [26].

### 2.2. Amount of DON, NIV, and Their 3-β-D-glucosides

**Figure 1:**
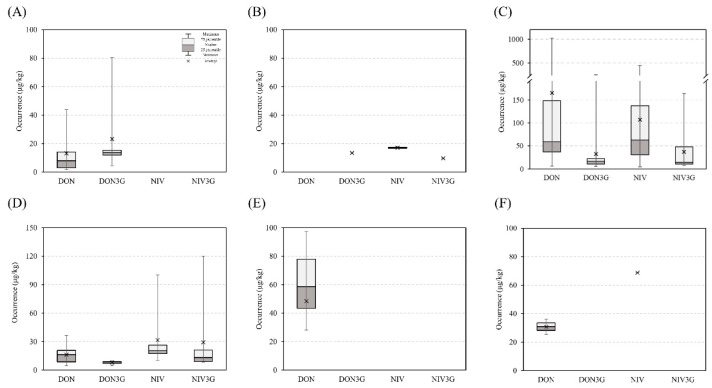
Occurrence of type B trichothecenes in six food groups (**A**) alcoholic beverages, (**B**) baby foods, (**C**) cereals and cereal-based products, (**D**) legumes and legume-based products, (**E**) noodles, (**F**) snacks.

The 506 food samples were categorized into the following six food groups: alcoholic beverages (A), baby foods (B), cereals and cereal-based foods (C), legumes and legume-based foods (D), noodles (E), and snacks (F). The amount of DON, DON3G, NIV, and NIV3G in these six groups is listed in Figure 1.

The six groups were further categorized into 37 subgroups (Table 2). Of the 506 samples, 13% were DON positive (mean: 101.9 μg kg^−1^, range: 2.0–1018.4 μg kg^−1^) and 8% were positive for DON3G (mean: 22.9 μg kg^−1^, range: 4.5–93.6 μg kg^−1^). Furthermore, 12% were positive for NIV (mean: 77.1 μg kg^−1^, range: 4.6–370.8 μg kg^−1^) and 5% were NIV3G positive (mean: 33.4 μg kg^−1^, range: 7.6–250.6 μg kg^−1^). The amount of these toxins in baby foods and Korean rice wine has been published [26].

In group A (alcoholic beverages), the detection rate of DON and DON3G was 17.1% (13.3 μg kg^−1^) and 17.1% (23.3 μg kg^−1^), respectively; NIV and NIV3G were not detected (<LOD). DON was detected in one Korean rice wine sample (6.2 μg kg^−1^). In beer, the concentration of DON3G (23.3 μg kg^−1^) was higher than that of DON (14.7 μg kg^−1^). Our results are similar to those of Bryla et al. [13]. During the germination of malt, glucose possibly binds to DON, catalyzed by activated enzymes, such as glucosyltransferase [27]. In group B (baby foods), none of the four toxins was detected in milk-based baby foods. DON3G (13.5 μg kg^−1^), NIV (17.1 μg kg^−1^), and NIV3G (9.8 μg kg^−1^) were detected in cereal-based baby foods. Therefore, contaminating glucoside conjugates remained even after food processing, although the contamination level was low. Group C (cereal and cereal-based products) exhibited the highest contamination level compared with the other groups, with detection rates of 21.8%, 12.1%, 20.1%, and 8.6% for DON, DON3G, NIV, and NIV3G, respectively, and with contamination levels of 165.6, 32.5, 107.2, and 37.3 μg kg^−1^, respectively. The major contributors to the incidence of trichothecenes in this group were barley, foxtail millet, job’s tears, and sorghum, all of which were produced in Korea. Although DON naturally occurs more frequently than NIV, in this study, the NIV occurrence rate was observed to be similar to that of DON. This may be because the gene chemotype of the *Fusarium* species in Korean cereals has been reported to be mostly the NIV type [28,29]. Trichothecenes in cereals and cereal-based foods have been studied. In Korea, the detection rate of DON and NIV was 4–54% and their mean concentration was 4–190 μg kg^−1^; these results are similar to those estimated in our study [6,30]. However, the detection rate of DON and NIV in other countries was 14–100% and the mean concentration was 1–17754 μg kg^−1^, which are higher than those observed in our study [7,31,32,33,34]. In this study, DON, at a concentration of 1018 μg kg^−1^, was detected in one foxtail millet sample, which was slightly over the maximum permissible limit, according to the Korean Food Code (1000 μg kg^−1^). In group D (legume and legume-based products), the detection rate of DON, DON3G, NIV, and NIV3G was 11.8%, 8.1%, 11.8%, and 5.6%, respectively, and their concentration was 16.4, 8.1, 31.7, and 29.4 μg kg^−1^, respectively. The concentration of NIV was higher than that of DON in this group. In group E (noodles), only DON was detected at 48.6 μg kg^−1^. In group F (snacks), the detection rate of DON and NIV was 3.8% and 1.9%, respectively, and their concentration was 30.9 and 68.7 μg kg^−1^, respectively. Overall, higher amounts of DON and NIV were detected more in groups C and D, which contained raw materials or slightly processed foods, than the other groups that contained highly processed foods. Glucoside conjugates as free toxins showed the same pattern, and highly processed foods had lower amounts of glucoside conjugates [35,36].

### 2.3. Co-Occurrence

**Figure 2:**
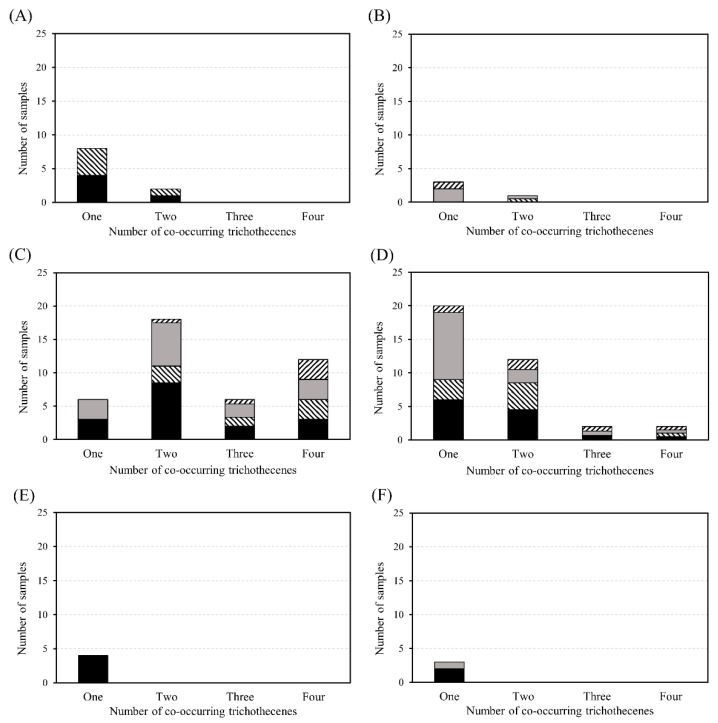
Co-occurrence of DON (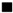), DON3G (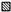), NIV (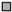), and NIV3G (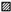) in commercial food groups. Panels (**A**), (**B**), (**C**), (**D**), (**E**), and (**F**) indicate alcoholic beverages, baby foods, cereals and cereal-based products, legumes and legume-based products, noodles, and snacks, respectively.

The co-occurrence of DON, NIV, and their glucosides is presented in Figure 2. DON, DON3G, NIV, and NIV3G were detected in 14 samples (5%); 12 samples were positive in group C and 2 samples were positive in group D. A total of 49% (33/68) of DON-contaminated samples were also found contaminated with DON3G, and the molar ratio of DON3G to DON was 10.5%. This result was higher than the reported co-occurrence ratio of about 30% in cereals [12,13]. Furthermore, 40% of NIV-contaminated samples were also contaminated with NIV3G, and the molar ratio of NIV3G to NIV was 17.9%. Group C presented a high co-occurrence (two or more toxins) ratio among the six food groups, possibly because this group included raw or only simple-processed foods, while the other groups included foods that were subjected to fermentation, heating, washing, and other physical and chemical processing.

### 2.4. Exposure to DON, NIV, and Their 3-β-D-Glucosides via Food Intake

In this study, we also estimated the potential exposure to these toxins through food intake. Food intake data included the mean and 95th percentile (an extreme daily intake) according to age. The mean body weight, according to age groups 1–2, 3–7, 8–12, 13–19, 20–50, and over 51 years, was 59.4, 12.3, 20.5, 39.4, 59.8, 65.8, and 61.6 kg, respectively [14]. The estimated daily intake (EDI) was calculated for four scenarios. The health risk characterization of each type B trichothecene was performed by dividing the calculated EDI by the TDI. In the present study, a group TDI was included with the same molar potency as NIV and DON, because NIV3G and DON3G are assumed to be hydrolyzed into NIV and DON, respectively, after ingestion [7,8,9].

The calculated EDI is presented in Table 3. The EDI values of DON, DON3G, NIV, and NIV3G through food intake in four different scenarios (lowest-to-highest exposure) were 0.019–0.102, 0.004–0.089, 0.007–0.094, and 0.002–0.095 μg kg^−1^ b.w. day^−1^, respectively. In addition, the EDI values of the combined intake of DON, DON3G, NIV, and NIV3G were 0.064, 0.090, 0.122, and 0.380 μg kg^−1^ b.w. day^−1^, respectively. The calculated values of %TDI, which is the percentage of TDI covered by the EDI, were 1.9–10.2% for DON, 0.4–8.9% for DON3G, 1.8–23.5% for NIV, and 0.5–23.8% for NIV3G in all the four scenarios. According to these results, DON, DON3G, NIV, and NIV3G showed exposure values below the JECFA and FSCJ established health-based guidance values and are thus unlikely to pose a health risk.

**Figure 3:**
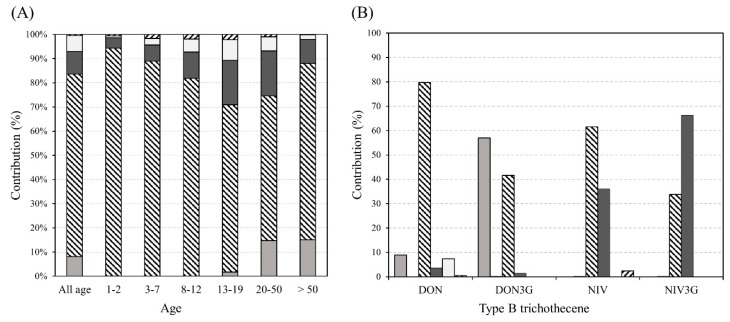
Exposure contribution of food groups in different age groups and type B trichothecene mycotoxins. (**A**) Exposure contribution of food groups, (**B**) exposure contribution of type B trichothecene (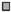: alcoholic beverages, 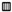: baby foods, 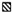: cereals and cereal based products, 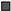: legumes and legumes-based products, 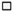: noodles, 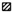: snacks).

The exposure contribution of the food groups is presented in Figure 3. Cereals and cereal-based food groups contributed the most (74.7%) to type B trichothecene exposure in all age groups. In age group 1–2 years, the highest contribution was through cereals and cereal-based food. Furthermore, as the age increased, the exposure contribution of other food groups increased. DON exposure in the cereals and cereal-based food group was the highest (79.7%), followed by the alcoholic beverage group (8.9%) and noodle group (7.3%). NIV exposure was the highest in the cereals and cereal-based food group (61.6%) and in the legumes and legume-based food group (36%). DON3G exposure was the highest in the alcoholic beverage group (57%), whereas NIV3G exposure was the highest in the legumes and legume-based food group (66.2%). These results suggest that cereals and cereal-based food contribute the most to DON and NIV exposure.

The estimated exposure of the glucoside conjugates in the present study was somewhat overestimated, because the study assumed that glucoside conjugates are 100% hydrolyzed and that their toxicity equals the potential toxicity of free toxins in the human gut. Thus, to accurately estimate the risk of glucoside conjugates, further evidence of the hydrolysis, absorption, and synergistic effects of glucoside conjugates in the mammalian gastrointestinal tract is needed.

## 3. Conclusions

To the best of our knowledge, this is the first study on the detection of major type B trichothecenes, DON, NIV, and their 3-β-D-glucoside conjugates, in food products marketed in Korea and the first evaluation of the exposure of the Korean population to these toxins. We analyzed DON, NIV, and their glucosides in 506 foods, which were categorized into six groups using a validated high-performance liquid chromatography (HPLC) method. In these foods, DON, DON3G, NIV, and NIV3G were detected at rates of 13% (101.9 μg kg^−1^), 8% (22.9 μg kg^−1^), 12% (77.1 μg kg^−1^), and 5% (33.4 μg kg^−1^), respectively. The glucoside conjugate with free toxin was found to occur at the maximum rate, at 49%. The TDI% values of DON, DON3G, NIV, and NIV3G through food intake in four different scenarios were 1.9–10.2%, 0.4–8.9%, 1.8–23.5%, and 0.5–23.8%, respectively. Overall, our results indicate that the estimated exposure of the Korean population to type B trichothecenes is not hazardous. However, continuous monitoring and risk assessment of DON, NIV, and their glucoside conjugates are imperative. Furthermore, for more accurate estimation, the risk of exposure to glucoside conjugates and clear evidence of hydrolysis of these toxins in the mammalian intestine are required, and toxicity studies of co-exposure to glucosides and free toxins are needed.

## 4. Materials and Methods

### 4.1. Samples

A total of 506 various food products were randomly purchased from Korean markets (Anseong, Anyang, Seoul, and Uiwang areas) from 2017 to 2018. The samples were classified into the following six food groups: alcoholic beverages (*n* = 35), baby foods (*n* = 27), cereals and cereal-based foods (*n* = 174), legumes and legume-based foods (*n* = 161), noodles (*n* = 57), and snacks (*n* = 52). Alcohol beverages consisted of beer (*n* = 20) (domestic 55%, imported 45%) and Korean rice wine (*n* = 15) (domestic 100%); baby foods consisted of cereal-based baby foods (*n* = 16) (domestic 69%, imported 31%) and milk-based baby foods (*n* = 11) (domestic 64%, imported 36%); cereals and cereal-based foods consisted of white rice (*n* = 17) (domestic 100%), brown rice (*n* = 22) (domestic 100%), glutinous rice (*n* = 7) (domestic 100%), wheat (*n* = 13) (domestic 92%, imported 8%), maize (*n* = 12) (domestic 92%, imported 8%), oats (*n* = 11) (domestic 82%, imported 18%), barley (*n* = 15) (domestic 100%), foxtail millet (*n* = 11) (domestic 100%), sorghum (*n* = 12) (domestic 100%), job’s tears (*n* = 11) (domestic 100%), buckwheat (*n* = 5) (domestic 100%), scorched rice (*n* = 5) (domestic 100%), canned corn (*n* = 4) (imported 100%), barley tea (*n* = 6) (domestic 100%), and breakfast cereal (*n* = 23) (domestic 91%, imported 9%); legumes and legume-based foods consisted of soybeans (*n* = 15) (domestic 100%), red beans (*n* = 15) (domestic 100%), mungbeans (*n* = 10) (domestic 100%), peas (*n* = 10) (domestic 70%, imported 30%), soymilk (*n* = 11) (domestic 100%), soybean paste (*n* = 15) (domestic 100%), soy sauce (*n* = 15) (domestic 87%, imported 13%), gochujang (Korean red pepper paste) (*n* = 15) (domestic 100%), mixed paste (*n* = 15) (domestic 100%), chunjang (black soybean paste) (*n* = 15) (domestic 93%, imported 7%), cheonggukjang (fast-fermented bean paste) (*n* = 15) (domestic 100%), and tofu (*n* = 10) (domestic 100%); noodle foods consisted of noodles (*n* = 15) (domestic 83%, imported 27%), ramen (*n* = 22) (domestic 91%, imported 9%), spaghetti (*n* = 16) (imported 100%), and kalguksu (*n* = 4) (domestic 100%); and snack foods consisted of snacks (*n* = 37) (domestic 68%, imported 32%), biscuit-cookies (*n* = 11) (domestic 82%, imported 18%), and popcorn (*n* = 4) (domestic 50%, imported 50%).

The samples (0.5–1 kg) were collected, ground, homogenized, and stored in an aluminum zipper bag at −20 °C until further analyses. Before the analyses, the samples were thawed to room temperature (20 °C).

### 4.2. Chemicals and Reagents

High-performance liquid chromatography (HPLC) grade solvents (water, methanol (MeOH), acetonitrile (ACN), and other solvents were purchased from Burdick & Jackson (Morris Plains, NJ, USA). The immunoaffinity column (IAC) DON-NIV^WB^ used for pretreatment was purchased from VICAM (Milford, MA, USA). DON (100.5 μg mL^−1^) and NIV (101.1 μg mL^−1^) were purchased from Romer Labs (Tulln, Austria). DON3G (50.3 μg mL^−1^) was purchased from Sigma (St Louis, MO, USA). NIV3G was synthesized, purified, and provided to us by the University of Natural Resources and Life Sciences (BOKU), Vienna, Austria. Its identity and purity (>98%) were verified by nuclear magnetic resonance (NMR) and HPLC-UV measurements [37]. Working standard solutions of trichothecenes were prepared in ACN at a concentration of 10 μg mL^−1^ and stored at −20 °C.

### 4.3. Extraction and Purification of Samples

DON, NIV, and their glucoside conjugates were extracted and purified using the established and validated method of Lee et al. [26]. The extraction steps were different for solid and liquid samples. For solid samples, 25 g of sample was dissolved in 100 mL of 20% ACN, homogenized using a homogenizer (6200 rpm, for 5 min), transferred into a 50 mL conical flask, and centrifuged (20,000× *g*, for 20 min). The supernatant was diluted five times with water and then passed through a glass microfiber filter (GF/B). The filtrate (20 mL) was passed through the immunoaffinity column (IAC) by gravity (one drop/s). The IAC was washed with 20 mL of water and dried using a syringe. The eluent was washed with MeOH (2 mL) and then dried in a heating block (50 °C) under nitrogen. The residue was reconstituted in 1 mL of mobile phase and passed through a 0.2-µm polyvinylidene fluoride syringe filter. For liquid samples, 25 g of sample was sonicated in a beaker for 10 min to remove carbonic acid, followed by the addition of 100 mL of water as an extraction solvent. The samples were then processed as described above. All samples were analyzed in triplicates, and their recovery was estimated. HPLC injections were done in triplicates.

### 4.4. HPLC–UV Analysis

The HPLC analysis was conducted using the Agilent 1260 infinity series (Santa Clara, CA, USA), consisting of an autosampler (G1329B), quaternary pump (G1311C), thermostatic column compartment (G1316A), thermostat (G1330B), and UV detector (G1314F), set at a wavelength of 218 nm. The Supelco Ascentis Express C18 columns (2.7 μm particle size; 4.6 mm × 150 mm) (Bellefonte, PA, USA) were used for toxin separation and analysis, following 100 μL sample injections at 30 °C. The mobile phases were water (A), MeOH (B), and ACN (C). The following gradient was used: 0 min (A/B/C, 95:1:4 (v/v/v)), 5 min (A/B/C, 95:1:4 (v/v/v)), and 20 min (A/B/C, 75:12.5:12.5 (v/v/v)); the flow rate was 0.8 mL/min. The retention times for DON, DON3G, NIV, and NIV3G were 12.8, 13.6, 7.2, and 8.3 min, respectively.

### 4.5. Method Validation

The analysis method of DON, NIV, and their glucosides was validated in four matrices that represented different physical characteristics, namely, baby foods (solid-flour), soybean paste (solid-paste), sorghum (solid-colored), and Korean rice wine (liquid). The recovery analyses were conducted intraday in triplicates for two concentrations: 2 × LOQ and 5 × LOQ. The accuracy and precision were evaluated by the mean recovery rate and the relative standard deviation (RSD) of the triplicate samples.

### 4.6. Estimation of Dietary Exposure

The estimated dietary exposure to DON, NIV, and their glucoside conjugates was evaluated by calculating the estimated daily intake (EDI) using the following formula: Dietary exposure = ∑ (concentration of toxins in food × food consumption)/body weight (kg). To determine the mean concentration of toxins in foods, the lower bound (LB) and upper bound (UB) approaches were used. In the LB approach, the result was replaced with a zero value for all samples with concentrations below the limit of detection (LOD), whereas in the UB approach, the result was replaced with the LOD value for all samples with concentrations between the LOD and LOQ. Four different scenarios were used to estimate exposure, including (1) LB concentration × mean consumption, (2) UB concentration × mean consumption, (3) LB concentration × 95% consumption, and (4) UB concentration × 95% consumption. When the 95th percentile intake data were not available, the mean intake data were used to calculate the EDI (beer, job’s tears, maize, wheat, red beans, soymilk, and popcorn). To calculate the EDI, the guidelines on food intake and body weight, according to age, based on the Fifth Korean National Health and Nutrition Examination Survey [14], were used. For DON, the calculated EDI was compared with the TDI of 1 μg kg^−1^ b.w. day^−1^ [23]. The EDI of NIV was compared with the TDI of 0.4 μg kg^−1^ b.w. day^−1^ [21]. Glucoside conjugates were assumed to be 100% hydrolyzed in the human intestine and their EDIs were compared with the TDIs of the free toxins (DON and NIV).

## Figures and Tables

**Table 1:** Recovery of deoxynivalenol (DON), nivalenol (NIV), and their 3-β-D-glucosides in four different matrices. Limit of quantification (LOQ); limit of detection (LOD); relative standard deviation (RSD).

Toxin	Parameter	Matrix			
		Baby Food (Solid-Flour)	Soybean Paste (Solid-Paste)	Sorghum (Solid-Colored)	Korean Rice Wine (Liquid)
DON	LOD (μg kg^−^^1^)	3.9	1.4	1.4	1.5
	LOQ (μg kg^−1^)	11.7	4.3	4.1	4.4
	Recovery (%)				
	2LOQ	87.4	95.8	103.9	100.0
	5LOQ	84.7	96.7	87.9	104.7
	RSD (%)				
	2LOQ	9.3	3.4	3.4	3.4
	5LOQ	12.4	5.3	1.9	3.0
DON3G	LOD (μg kg^−1^)	5.3	1.5	1.4	1.5
	LOQ (μg kg^−1^)	16.1	4.6	4.1	4.5
	Recovery (%)				
	2LOQ	86.5	84.5	87.1	103.0
	5LOQ	82.9	89.7	86.5	96.2
	RSD (%)				
	2LOQ	0.3	5.0	5.8	2.8
	5LOQ	2.0	7.2	9.9	2.0
NIV	LOD (μg kg^−1^)	3.5	1.3	1.9	2.0
	LOQ (μg kg^−1^)	10.6	3.9	5.8	6.1
	Recovery (%)				
	2LOQ	103.3	85.4	98.2	95.0
	5LOQ	90.8	84.9	80.1	100.1
	RSD (%)				
	2LOQ	4.1	12.9	9.5	2.5
	5LOQ	7.2	4.3	6.3	2.4
NIV3G	LOD (μg kg^−1^)	4.6	1.2	1.0	1.1
	LOQ (μg kg^−1^)	13.8	3.5	3.1	3.3
	Recovery (%)				
	2LOQ	90.6	98.3	88.9	106.5
	5LOQ	88.2	94.1	80.6	105.8
	RSD (%)				
	2LOQ	1.5	2.6	6.1	2.5
	5LOQ	9.4	5.6	2.6	3.0

**Table 2:** Occurrence of DON, DON3G, NIV, and NIV3G in commercial foods.

Food Group ^1^/Subgroup	DON		DON3G		NIV		NIV3G	
	Range	Positive Mean	Range	Positive Mean	Range	Positive Mean	Range	Positive Mean
A								
Beer	2.0–43.9	14.7 (5/20)^2^	4.5–80.3	23.3 (6/20)	-^5^	0.0 (0/20)	-	0.0 (0/20)
Rice wine	6.2	6.2 (1/15)	-	0.0 (0/15)	-	0.0 (0/15)	-	0.0 (0/15)
B								
Baby formula^3^	-	0.0 (0/16)	13.5	14.0 (1/16)	16.5–17.9	17.0 (3/16)	9.8	10.0 (1/16)
Baby formula^4^	-	0.0 (0/11)	-	0.0 (0/11)	-	0.0 (0/11)	-	0.0 (0/11)
C								
Barley	11.7–286.0	75.8 (5/15)	18.0–20.6	19.3 (2/15)	17.3–229.6	90.2 (6/15)	10.4–110.3	60.3 (2/15)
Barley tea	-	0.0 (0/6)	-	0.0 (0/6)	-	0.0 (0/6)	-	0.0 (0/6)
Breakfast cereal	5.6–88.6	44.0 (4/23)	5.0–17.0	9.7 (4/23)	-	0.0 (0/23)	-	0.0 (0/23)
Brown rice	-	0.0 (0/22)	-	0.0 (0/22)	47.4	47.4 (1/22)	-	0.0 (0/22)
Buckwheat	-	0.0 (0/5)	-	0.0 (0/5)	-	0.0 (0/5)	-	0.0 (0/5)
Canned corn	-	0.0 (0/4)	-	0.0 (0/4)	-	0.0 (0/4)	-	0.0 (0/4)
Foxtail millet	18.8–1018.4	214.8 (6/11)	56.2–93.6	74.9 (2/11)	27.4–370.8	151.8 (5/11)	56.2–164.1	110.2 (2/11)
Glutinous rice	-	0.0 (0/7)	-	0.0 (0/7)	-	0.0 (0/7)	-	0.0 (0/7)
Job’s tears	22.6–751.4	306.3 (9/11)	6.6–26.4	15.9 (7/11)	12.6–337.6	133.8 (10/11)	7.6–39.2	14.6 (7/11)
Maize	445.0	445.0 (1/12)	56.3	56.3 (1/12)	51.3	51.3 (1/12)	-	0.0 (0/12)
Oat	-	0.0 (0/11)	-	0.0 (0/11)	23.5	23.5 (1/11)	33.5	33.5 (1/11)
Scorched rice	-	0.0 (0/5)	-	0.0 (0/5)	-	0.0 (0/5)	-	0.0 (0/5)
Sorghum	18.9–711.7	119.0 (12/12)	10.4–43.4	18.8 (5/12)	4.6–145.8	54.3 (11/12)	11.5–14.8	13.2 (2/12)
Wheat	-	0.0 (0/13)	-	0.0 (0/13)	211.9	211.9 (1/13)	250.6	250.6 (1/13)
White rice	-	0.0 (0/17)	-	0.0 (0/17)	-	0.0 (0/17)	-	0.0 (0/17)
D								
Cheonggukjang	-	0.0 (0/15)	7.5	7.5 (1/15)	-	0.0 (0/15)	-	0.0 (0/15)
Chunjang	19.0–36.5	23.0 (7/15)	7.1–9.9	8.0 (8/15)	83.8	83.8 (1/15)	54.5	54.5 (1/15)
Gochujang	4.7–32.2	16.0 (4/15)	6.7	6.7 (1/15)	16.0–89.9	29.0 (9/15)	8.5–120.2	34.0 (5/15)
Mixed paste	7.7–8.2	8.0 (2/15)	4.7	4.7 (1/15)	15.9–100.6	35.0 (6/15)	12.8–16.3	14.0 (3/15)
Mungbean	-	0.0 (0/10)	-	0.0 (0/10)	-	0.0 (0/10)	-	0.0 (0/10)
Pea	-	0.0 (0/10)	-	0.0 (0/10)	-	0.0 (0/10)	-	0.0 (0/10)
Red bean	4.9–9.6	8.0 (3/15)	9.4	9.4 (1/15)	17.2	17.2 (1/15)	-	0.0 (0/15)
Soybean	15.7	15.7 (1/15)	9.6	9.6 (1/15)	-	0.0 (0/15)	-	0.0 (0/15)
Soybean paste	11.1–16.5	14.0 (2/15)	-	0.0 (0/15)	-	0.0 (0/15)	-	0.0 (0/15)
Soymilk	-	0.0 (0/11)	-	0.0 (0/11)	10.6	10.6 (1/10)	-	0.0 (0/11)
Soy sauce	-	0.0 (0/15)	-	0.0 (0/15)	21.8	21.8 (1/15)	-	0.0 (0/15)
Tofu	-	0.0 (0/10)	-	0.0 (0/10)	-	0.0 (0/10)	-	0.0 (0/10)
E								
Kalguksu	-	0.0 (0/4)	-	0.0 (0/4)	-	0.0 (0/4)	-	0.0 (0/4)
Noodle	10.1	10.1 (1/15)	-	0.0 (0/15)	-	0.0 (0/15)	-	0.0 (0/15)
Ramen	28.3–97.3	61.4 (3/22)	-	0.0 (0/22)	-	0.0 (0/22)	-	0.0 (0/22)
Spaghetti	-	0.0 (0/16)	-	0.0 (0/16)	-	0.0 (0/16)	-	0.0 (0/10)
F								
Biscuit, cookie	-	0.0 (0/11)	0.0	0.0 (0/11)	0.0	0.0 (0/11)	-	0.0 (0/11)
Popcorn	-	0.0 (0/4)	0.0	0.0 (0/4)	68.7	68.7 (1/4)	-	0.0 (0/4)
Snack	25.7–36.1	30.9 (2/37)	0.0	0.0 (0/37)	0.0	0.0 (0/37)	-	0.0 (0/37)
Total	2.0–1018.4	101.9 (68/506)	4.5–93.6	22.9 (41/506)	4.6–370.8	77.1 (59/506)	7.6–250.6	33.4 (25/506)

^1^. A: alcoholic beverages, B: baby foods, C: cereals and cereal based products, D: legumes and legumes-based products, E: noodles, F: snacks. ^2^ Values in parentheses indicate the number of positive/total samples, ^3^ Cereal-based baby food. ^4^ Milk-based baby food. ^5^ Not detected (below LOD).

**Table 3:** Estimation of daily intake and exposure assessment of DON, NIV, and their 3-β-D-glucosides in commercial foods through mean or 95 percentile food consumptions.

Parameter	DON	DON3G	∑ (DON + DON3G	NIV	NIV3G	∑ (NIV + NIV3G)
Occurrence (μg kg^−1^)						
Lower bound	13.7	1.9	7.8	8.8	1.6	5.2
Upper bound	17.3	6.9	12.1	12.9	7.6	10.3
Food consumption (g kg^−1^ b.w. day^−1^)						
Mean intake	0.00–160.06					
95% intake	0.00–347.73					
EDI (μg kg^−1^ b.w. day^−1^)/%TDI^1^						
Scenario 1^2^	0.019/1.9	0.004/0.4	0.023/2.3	0.007/1.8	0.002/0.5	0.009/2.3
Scenario 2	0.046/4.6	0.006/0.6	0.052/5.2	0.029/7.2	0.009/2.2	0.038/9.4
Scenario 3	0.038/3.8	0.032/3.2	0.070/7.0	0.030/7.4	0.032/7.9	0.062/15.3
Scenario 4	0.102/10.2	0.089/8.9	0.191/19.1	0.094/23.5	0.095/23.8	0.189/47.3

^1^ The percentage of tolerable daily intake (TDI) (1.0 μg kg^−1^ b.w. day^−1^ for DON and DON3G, 0.4 μg kg^−1^ b.w. day^−1^ for NIV and NIV3G) covered by the estimated daily intake (EDI). ^2^Scenario 1: Lower bound (LB) concentration × mean consumption; Scenario 2: Upper bound (UB) concentration × mean consumption; Scenario 3: LB concentration × 95% consumption; and Scenario 4: UB concentration × 95% consumption.

## Abbreviations

Deoxynivalenol (DON); nivalenol (NIV); deoxynivalenol-3-β-D-glucoside (DON3G); nivalenol-3-β-D-glucoside (NIV3G); limit of detection (LOD); limit of quantification (LOQ); estimated daily intake (EDI); tolerable daily intake (TDI); lower bound (LB); upper bound (UB); high performance liquid chromatography (HPLC); methanol (MeOH); acetonitrile (ACN); immunoaffinity column (IAC); relative standard deviation (RSD).
